# Supplementary material for: Genome-Wide Association Study of Feed Efficiency Related Traits in Ducks
Source: Animals (Basel). 2022 Jun 13;12(12):1532. doi: 10.3390/ani12121532 (PMC9219419; doi:10.3390/ani12121532)
Supplement: Supplementary file 1 [file animals-12-01532-s001.zip › animals-1716939-supplementary.pdf]

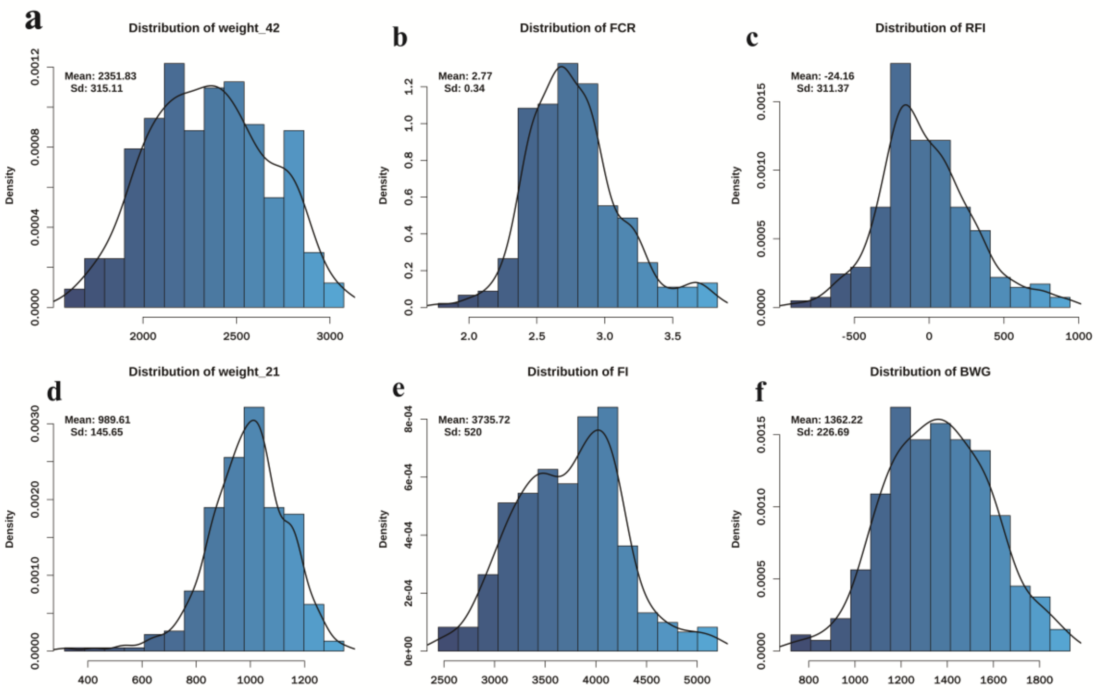

Figure S1. Frequency distribution of the adjusted phenotypes of (a) weight at 42 days of age, (b) feed conversion ratio (FCR), (c) residual feed intake (RFI), (d) weight at 21 days of age, (e) feed intake (FI), (f) body weight gain (BWG).

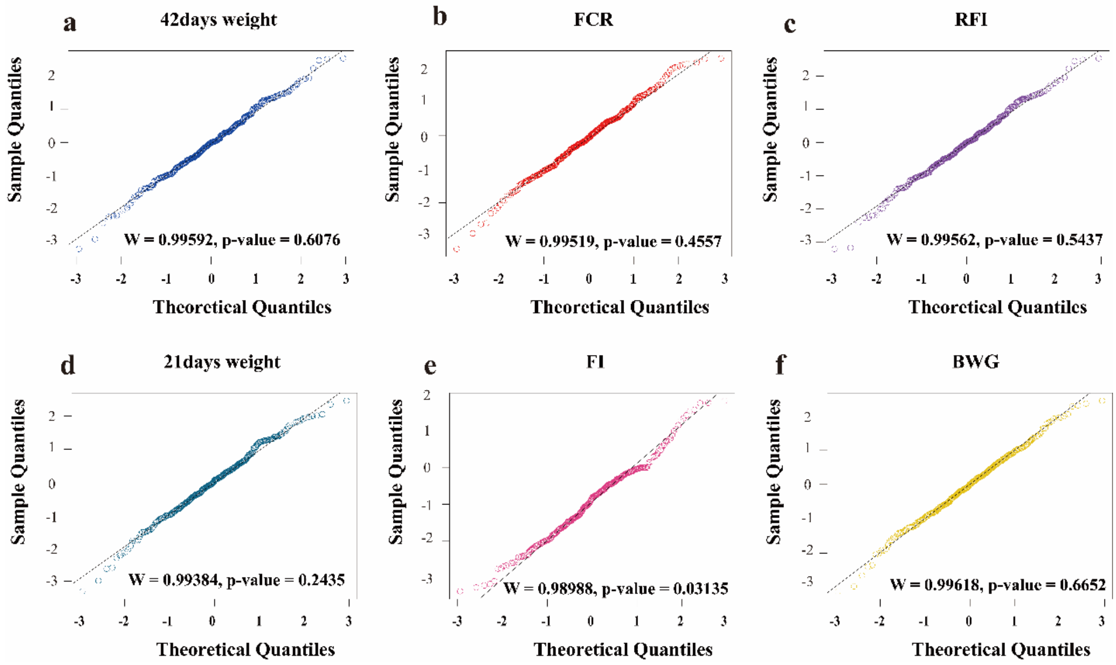

Figure S2. Q-Q plot for trait distribution visualization. (a) weight at 42 days of age, (b) feed conversion ratio (FCR), (c) residual feed intake (RFI), (d) weight at 21 days of age, (e) feed intake (FI), (f) body weight gain (BWG)

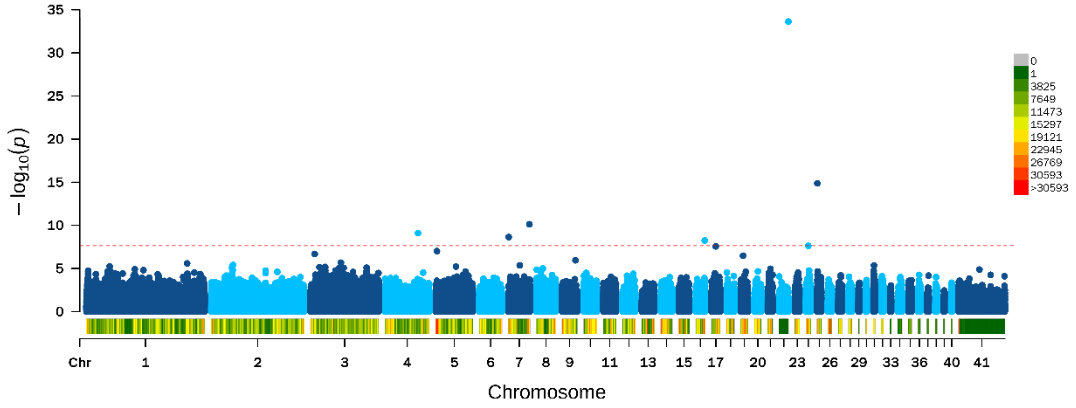

Figure S3. Manhattan plot of BWG.

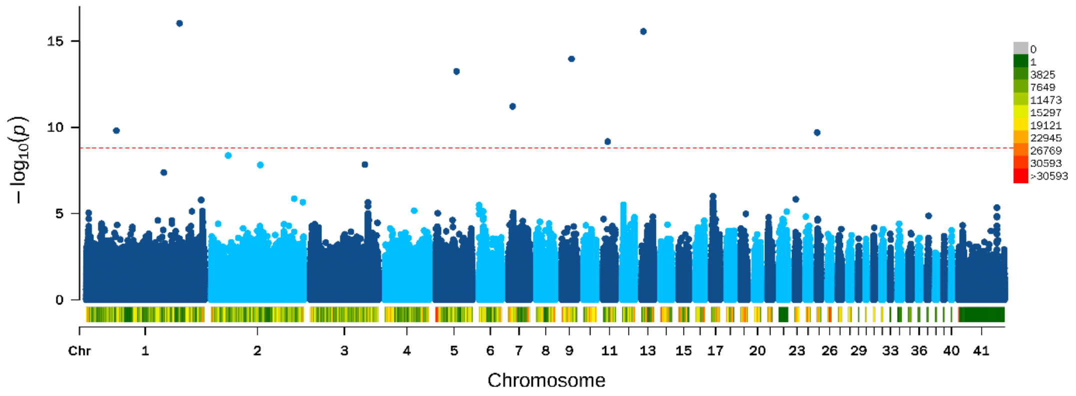

Figure S4. Manhattan plot of weight at 21 days of age.

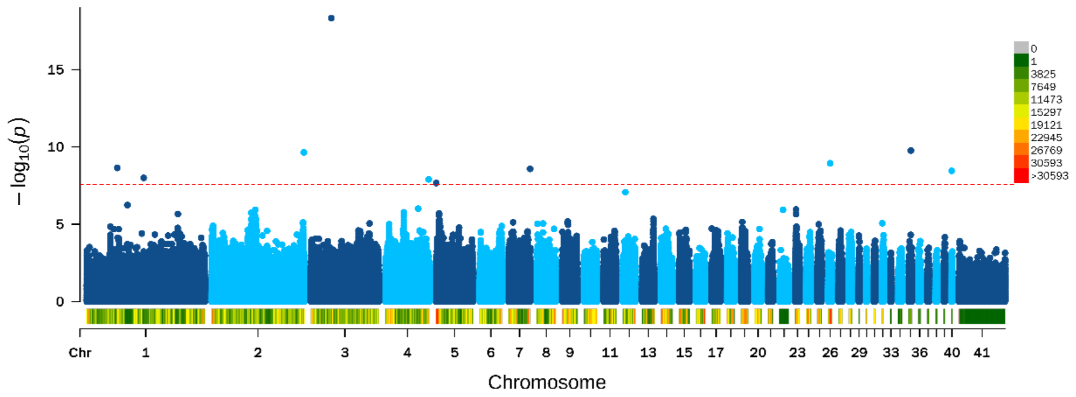

Figure S5. Manhattan plot of weight at 42 days of age.

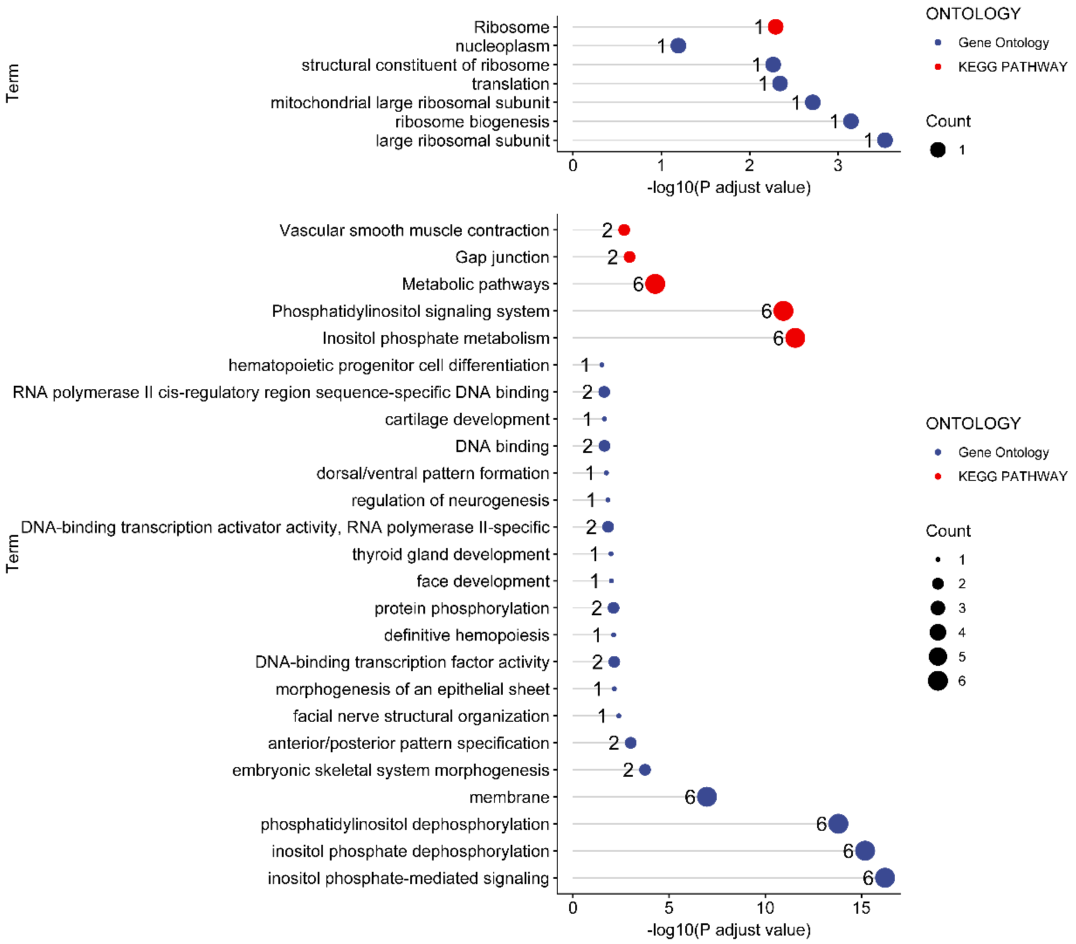

Figure S6. GO and KEGG enrichment of candidate genes of weight at 21 days (top) of age and BWG (bottom). Red and blue colored ribbons represent GO terms and KEGG pathways, respectively.

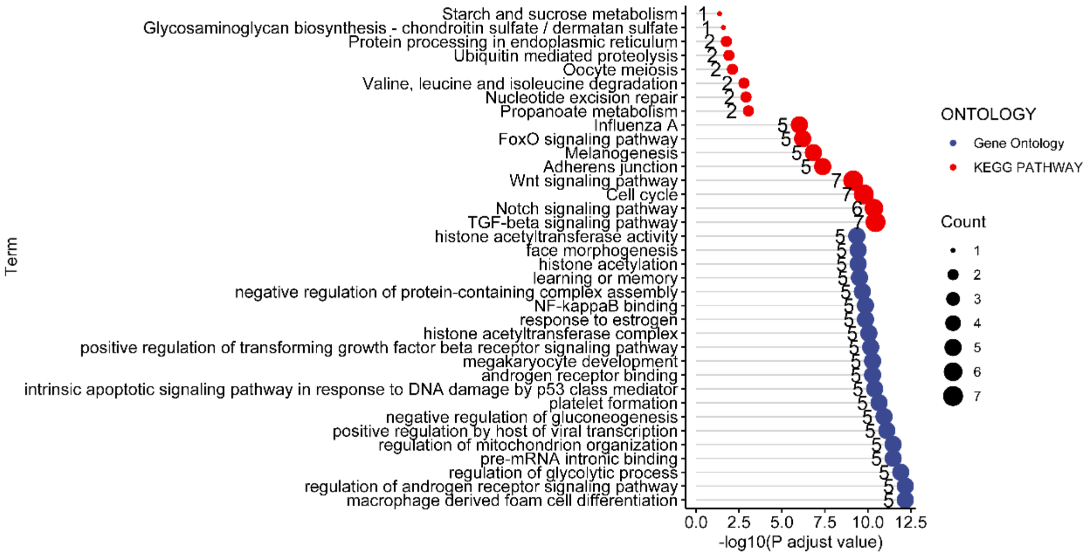

Figure S7. GO and KEGG enrichment of candidate genes of weight at 42 days of age. Red and blue colored ribbons represent GO terms and KEGG pathways, respectively.

Table S1. The information of all significant SNPs of each trait in duck.

Phenotype	CHROM	POS	*P* value	Gene
FCR	15	18051836	4.00E-09	EDIL3
	41	7575528	2.57E-09	COX7C,ERCC4
	41	7575530	1.42E-09	COX7C,ERCC4
	41	7597678	9.26E-10	COX7C, ERCC4
FI	9	2718579	1.14E-08	OTOL1, SI
	9	2718583	6.33E-09	OTOL1, SI
	9	2719545	1.63E-08	OTOL1, SI
RFI	1	87418567	8.31E-07	LSAMP, GAP43
	1	87426102	7.07E-07	LSAMP, GAP43
	1	87427326	7.04E-07	LSAMP, GAP43
	1	87429148	5.31E-07	LSAMP, GAP43
	1	87432872	6.58E-07	LSAMP, GAP43
	1	87435586	6.37E-07	LSAMP, GAP43
	1	87435687	7.16E-07	LSAMP, GAP43
	1	87439068	8.50E-07	LSAMP, GAP43
	1	87441494	8.15E-07	LSAMP, GAP43
	1	87451548	7.01E-07	LSAMP, GAP43
	1	87454544	4.57E-07	LSAMP, GAP43
	1	87455423	6.36E-07	LSAMP, GAP43
	1	87455452	6.69E-07	LSAMP, GAP43
	1	87455480	3.63E-07	LSAMP, GAP43
	1	87455495	5.75E-07	LSAMP, GAP43
	1	87456520	8.00E-07	LSAMP, GAP43
	1	87458582	8.51E-07	LSAMP, GAP43
	1	87505282	7.12E-07	LSAMP, GAP43
	1	87513379	4.52E-07	LSAMP, GAP43
	1	87514827	6.88E-07	LSAMP, GAP43
	1	87515821	7.36E-07	LSAMP, GAP43
	1	87518086	7.57E-07	LSAMP, GAP43
	1	87542599	6.06E-07	LSAMP, GAP43
	1	87542603	8.77E-07	LSAMP, GAP43
	1	87554583	9.48E-07	LSAMP, GAP43
	1	87561219	7.71E-07	LSAMP, GAP43
	1	87644532	8.81E-07	LSAMP, GAP43
	1	87651051	9.37E-07	LSAMP, GAP43
	3	108390147	5.79E-07	B3GNT8, ENSAPLG00020009332
	4	3989464	4.44E-07	FAM241A, RAP1GDS1,
	4	8809615	2.16E-07	UNC5C, SUCO,
	8	10355591	9.17E-07	ENSAPLG00020001335, ENSAPLG00020001341,
	8	10393777	7.25E-07	ENSAPLG00020001335, ENSAPLG00020001341,
	8	10393782	7.25E-07	ENSAPLG00020001335, ENSAPLG00020001341,

	8	11029074	4.56E-07	ENSAPLG00020001335, ENSAPLG00020001341,
	17	6214798	2.24E-07	GSTT1
BW21	1	53355120	1.54E-10	CCDC59, PPFIA2
	1	164362184	9.26E-17	TBC1D4
	5	36777337	5.69E-14	SUSD6
	7	7746590	6.09E-12	PNLIPRP2
	9	17111313	1.07E-14	NGEF
	11	7920952	6.67E-10	GABRE
	13	3216456	2.75E-16	ENSAPLG00020002037, CBLN1
	25	711933	1.99E-10	MRPL10
BW42	1	53921663	2.27E-09	EP300
	1	100402999	9.98E-09	ENSAPLG00020000057, GBE1
	2	162157988	2.26E-10	SPRY2, NDFIP2
	3	36210776	4.72E-19	BCKDHB
	4	76022028	1.25E-08	IL1RAP
	5	148077	2.13E-08	ENSAPLG00020011494, Mdga2
	7	37584853	2.59E-09	RNLS, ENSAPLG00020005166
	26	3399856	1.13E-09	FOXP4
	35	3704565	1.72E-10	ENSAPLG00020010580
	40	243346	3.51E-09	ENSAPLG00020009757
BWG	4	57724933	7.95E-10	TBC1D19
	7	398021	2.28E-09	INPP5A
	7	36914107	7.39E-11	PRKG1
	16	15666074	5.70E-09	ENSAPLG00020007801
	22	16007182	2.32E-34	ENSAPLG00020014612, RPL37
	25	437309	1.35E-15	HOXB3, HOXB2
